# Impact of Salt Reduction on Medical Expenditure for Hypertension in Japan: National and Subnational Simulation Models

**DOI:** 10.3390/nu18060933

**Published:** 2026-03-16

**Authors:** Nobuo Nishi, Takehiro Sugiyama, Sayuri Goryoda, Yutaka Takahashi, Katsuyuki Miura, Nayu Ikeda

**Affiliations:** 1 Graduate School of Public Health, St. Luke’s International University, Chuo 104-0045, Japan; 2 Diabetes and Metabolism Information Center, National Institute of Global Health and Medicine, Japan Institute for Health Security, Shinjuku 162-8655, Japan; sugiyama.t@jihs.go.jp; 3 Department of Health Services Research, Institute of Medicine, University of Tsukuba, Tsukuba 305-8575, Japan; 4 Graduate School of Agricultural Sciences, Yamagata University, Tsuruoka 997-8555, Japan; goryoda@tds1.tr.yamagata-u.ac.jp; 5 School of Commerce, Senshu University, Chiyoda 101-8425, Japan; takahasi@isc.senshu-u.ac.jp; 6 NCD Epidemiology Research Center, Shiga University of Medical Science, Otsu 520-2192, Japan; miura@belle.shiga-med.ac.jp; 7 Center for Nutritional Epidemiology and Policy Research, National Institute of Health and Nutrition, National Institutes of Biomedical Innovation, Health and Nutrition, Settsu 566-0002, Japan; ikedan@nibn.go.jp

**Keywords:** hypertension, Japan, medical expenditure, salt reduction, system dynamics

## Abstract

**Objective:** Excessive salt intake affects blood pressure. Thus, monitoring of salt intake is vital in the aging population. In this study, we examined the impact of salt intake reduction on medical expenditure for hypertension in Japan. **Methods:** System dynamics models for Japan and its 47 prefectures were developed using sub-models of salt intake, population, hypertension, and treatment costs. Co-flows of foods with high and low salt content and people with high and low salt intakes were built to calculate the daily salt intake. Aging chains of 10-year age groups from 40 to 79 years were built by sex for the population and people with hypertension (PwH). The outpatient treatment costs for hypertension were also calculated. The model parameters were calibrated using national statistics from 2012 to 2023, and prefectural models were developed to address the gaps in the national data for 2012. The simulated changes were compared across scenarios. **Results:** In the base run from 2012 to 2040, a reduction of 46.3% of high-salt foods and 33.1% of people with high salt intake would make a 13.9% reduction in daily salt intake from 10.1 g/day to 8.7 g/day. When salt intake is reduced to 6.9 g/day in 2040, PwH and treatment costs decrease by 2.3% and 2.0% in men and by 8.8% and 8.3% in women, respectively. The prefectural models exhibited the simulated changes. **Conclusions:** Our models suggest that reducing salt intake will lead to a decrease in the number of PwH and outpatient treatment costs by 2040.

## 1. Introduction

The daily salt intake among Japanese adults has decreased, according to the National Health and Nutrition Survey (NHNS) [1,2]. However, it remains high at 9.6 g in 2024 [3], exceeding the recommendations of the World Health Organization (WHO) (<5 g) [4] and the Dietary Reference Intakes for Japanese (<7.5 g for men and <6.5 g for women) [5]. Approximately 70% of dietary salt intake in Japan is attributable to common seasonings such as soy sauce and soybean paste [2]. Therefore, it is important to change not only individual dietary behaviors but also the food environment. Recently, various initiatives to reduce salt intake have been implemented in Japan, including the Strategic Initiative for a Healthy and Sustainable Food Environment and Health Japan 21 (Third Term) [6,7,8].

Excessive dietary salt intake increases blood pressure; thus, hypertension should be appropriately managed to prevent cardiovascular disease [9]. The prevalence of hypertension has decreased but is still approximately 40–60% in Japan [10]. Sixteen million people will receive treatment for hypertension in 2023 [11], with 1.7 trillion yen spent on hypertension treatment, accounting for 3.6% of the total national medical expenditures of 46.7 trillion yen in 2022 [12]. However, the control rate of hypertension is low, at 20–30% in Japan compared with 50–60% in the United States [10]. Effective hypertension control should be enforced in combination with the prevention of hypertension, including salt reduction.

Simulation studies are useful for demonstrating the effectiveness of public health interventions for chronic diseases [13]. Systems dynamics has been used to model chronic disease prevention [14]. The Prevention Impacts Simulation Model (PRISM) [15,16] simulates upstream and downstream strategies for preventing cardiovascular diseases in the United States, and its simulator is available to local governments to select policy interventions and view the resulting changes (https://prism-simulation.cdc.gov/app/cdc/prism/#/ (accessed on 2 February 2026)). A systematic review [14] included one Japanese article that modeled prevention in diabetic nephropathy [17]. System dynamics has also been used to model the health and economic effects of salt-reduction interventions in Japan [18]. Based on these studies using system dynamics in national models, we developed national and subnational models to examine the impact of salt reduction on medical expenditure for hypertension.

## 2. Materials and Methods

### 2.1. Development of a National Model

A system dynamics model for Japan was constructed using four sub-models: salt intake, population, hypertension, and treatment costs. The salt intake sub-model was developed for a fixed hypothetical population aged ≥20 years with a fixed number of foods. The rationale for using the fixed hypothetical population was to apply the common parameter to subnational models with modifications to other parameters in the following sub-model. For simplicity, the total number of foods and the total population were selected arbitrarily, as these numbers were allowed to have decimal points. Sub-models of population, hypertension, and treatment costs were based on national statistics as reference data, and the model parameters were calibrated for each sub-model in a stepwise manner. The model was developed using Vensim Professional 10.2.0 (Ventana Systems Inc., Harvard, MA, USA) [19]. The unit of time was years, and the initial time, final time, and time step were set as 2012, 2040, and 0.0625 years, respectively.

#### 2.1.1. Salt Intake Sub-Model

**Figure 1:**
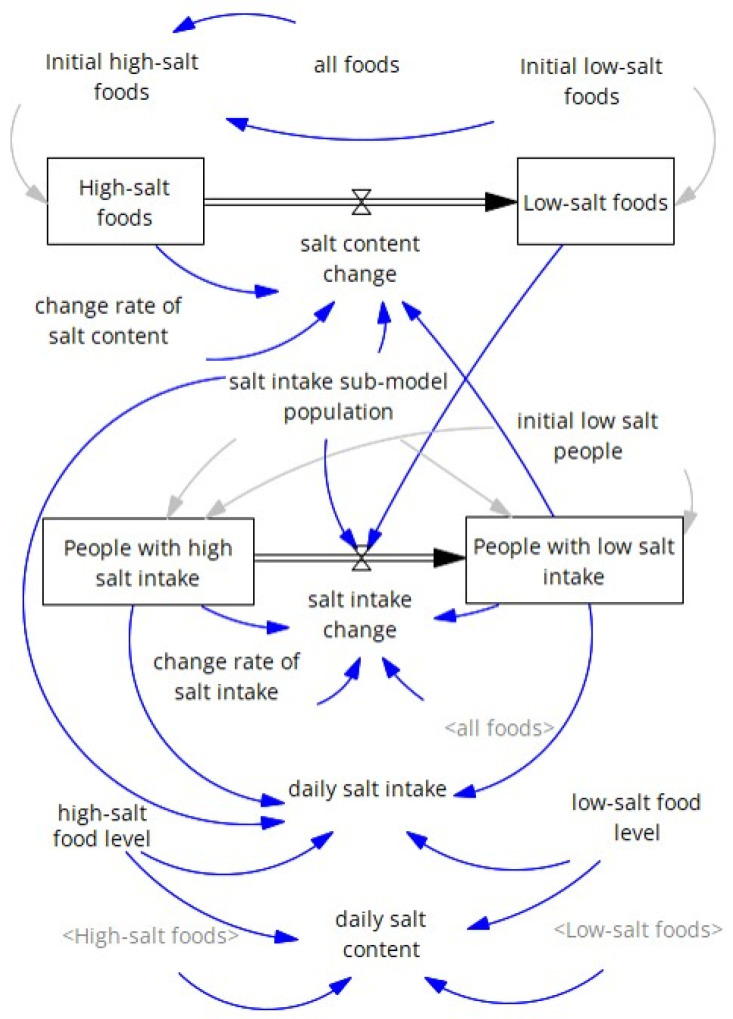
Structure of the salt intake sub-model. Rectangles represent stocks. Pipes with double arrows indicate flows that change stock values. Valves located at the center of the pipes regulate flow rates. Blue arrows represent causal links between elements. Gray arrows represent links for stocks that receive their initial values. Gray texts denote shadow variables that appear elsewhere in the model.

Based on the fixed hypothetical population and the fixed number of foods, the salt intake sub-model comprised two stock-and-flow diagrams of co-flows (Figure 1) [20]. One had two food stocks with high and low salt content, with a flow between them. The other had two populations with high and low salt intake, with a flow between them. These flows were directed from the left (high) to the right (low) to reflect a long-term decreasing trend in daily salt intake [21]. The stock of low-salt foods was linked to changes in salt intake among people, and the stock of people with low salt intake was linked to changes in food salt content. On average, high- and low-salt foods were assumed to contain 14 and 6 g of salt, respectively. These values were chosen to approximate the mean daily salt intake of 10.4 g ± 4.2 g (standard deviation) among people aged ≥20 years in the NHNS in 2012 [21]. The total number of foods was fixed at 1000, and 10% were initially assumed to be low in salt in 2012. The daily salt content was calculated as a weighted mean of 14 g and 6 g based on the proportions of high-salt foods and low-salt foods, respectively. The rate of change in the flow from the stock of high-salt foods to that of low-salt foods (“change rate of salt content” in Figure 1) was set at 0.04/year. The total population was fixed at 100,000, and the initial proportion with low salt intake was determined via optimization. In this study, we consistently performed optimization-based calibration to minimize the sum of absolute differences between simulated results and reference data using the Powell method as an unconstrained optimizer. We confirmed the absence of multiple identifiability by limiting parameter variation within a reasonable range consistent with existing knowledge [22]. The change rate of flow from the stock of people with a high salt intake to the stock of those with a low salt intake (“change rate of salt intake” in Figure 1) was also determined by optimization. These model parameters were calibrated to the daily salt intake of participants aged ≥20 years in NHNS, annually from 2012 to 2023; surveys in 2020 and 2021 were canceled due to COVID-19 [21]. The daily salt intake was calculated as a weighted mean of 14 g and 6 g based on the proportions of people with high and low salt intake, respectively. Daily salt intake by sex was set to differ consistently from that of both sexes combined, with gaps between the sexes combined and each sex in daily salt intake for participants aged ≥20 years in the NHNS in 2012 [21].

#### 2.1.2. Population Sub-Model

**Figure 2:**
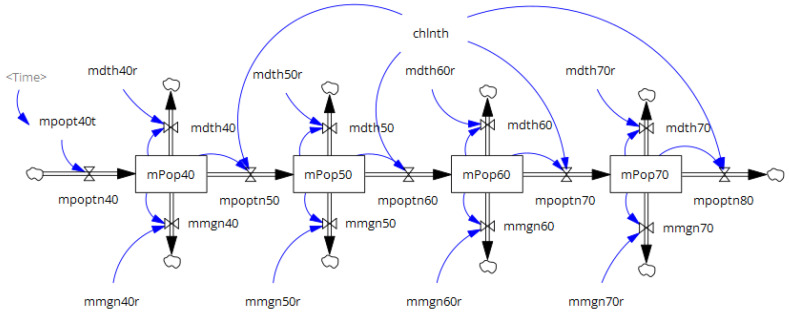
Structure of the population sub-model for men. Rectangles represent stocks. Clouds represent stocks outside the boundary of the system. Pipes with double arrows indicate flows that change stock values. Valves located at the center of the pipes regulate flow rates. Blue arrows represent causal links between elements. Gray texts denote shadow variables that appear elsewhere in the model.

The population sub-model comprises two stock-and-flow diagrams of the aging chains for men and women (Figure 2) [20]. Each aging chain has four stocks in the 10-year age group from 40 to 79 years, with the respective population in 2012 as its initial value [23]. The transition rates for the flows between stocks were set to 0.1/year, corresponding to an inverse of 10 years. Individuals aged 39 years enter inflows into stocks aged 40–49 years by sex. The number of people aged 39 years was obtained from previous years (2012–2019), and the number of people aged 18–38 years in 2019 was projected to be 39 years for 2020–2040 [23]. The 2020 statistics were not used to avoid incorporating abrupt changes attributable to COVID-19. In addition to the aging flow, each stock experienced outflows due to mortality and migration, which were calculated by multiplying the stock’s population by its respective parameters. These model parameters were calibrated to the population and sex- and age-group-specific mortality data annually from 2012 to 2019 [23,24]. The inverse of the standard error of the reference data was used as the calibration weight.

#### 2.1.3. Hypertension Sub-Model

The hypertension sub-model comprises two aging chains of people with hypertension (PwH): men and women. The basic structure of the aging chain of this sub-model is the same as that of the population sub-model. For the initial values of each stock, the number of PwH by sex and age group was calculated by multiplying the respective population and proportions of hypertension (systolic blood pressure ≥ 140 mmHg and/or diastolic blood pressure ≥ 90 mmHg) regardless of antihypertensive medication in 2012 using the NHNS data with permission from the Ministry of Health, Labor and Welfare (MHLW). For inflows into stocks with 40–49 years, the numbers of PwHs by sex were calculated using the same multiplication as for the initial values of each stock, based on the NHNS data from 2012 to 2023 (excluding 2020 and 2021), and the 2023 data were used continuously through 2040. In addition to aging, each stock exhibited an inflow of developing hypertension and an outflow of deaths. The inflow of individuals developing hypertension by sex and age group was calculated as the product of the proportion of individuals with high salt intake from the salt intake sub-model, the corresponding population size from the population sub-model, and the parameters. The outflows of deaths by sex and age group were calculated as 1.6 times the deaths from the respective stocks, based on findings from a nationally representative cohort of the Japanese population [25]. The parameters for the inflows were calibrated to the respective stocks of the product of the population and the proportions of PwH by sex and age group in the NHNS annually from 2012 to 2023 (excluding 2020 and 2021) [21], using the weights explained above.

#### 2.1.4. Treatment Cost Sub-Model

The treatment cost sub-model comprised no stock-and-flow diagrams but was built on stocks for the numbers of PwH by sex and age group from the hypertension sub-model. Treatment costs were calculated as the product of the number of PwH and the per-person outpatient treatment cost for hypertension. The outpatient treatment cost for hypertension per person by sex and age group was estimated by calibrating the annual outpatient medical expenditure for hypertension from 2012 to 2019 in the Medical Insurance Benefits Survey [26], using the weights described above.

### 2.2. Development of Subnational Models

Subnational models for the 47 prefectures were developed separately using the same sub-models and parameters as the national model. The only exception was that the model parameters for the outflows of deaths and migration in the population sub-model were calibrated to population and death counts in each prefecture, by sex and age group, annually from 2012 to 2019 [20,21], using the weights described above.

In the salt intake sub-model, a gap in daily salt intake for the participants aged ≥20 years of the NHNS in 2012 [21] between each prefecture and the whole country was applied to the salt content (14 g and 6 g) of high- and low-salt foods to calculate prefecture-specific daily salt intake. In the hypertension sub-model, the ratio of the 2012 prefecture-level proportion of participants aged 40–79 years with hypertension to the national proportion, based on NHNS individual-level data with MHLW permission, was used to calculate prefecture-specific numbers of PwH.

Relevant parameters of the prefectural models in Vensim Professional 10.2.0, which incorporated the national model, were transferred to each array using Stella Architect version 4.0 (ISEE Systems, inc., Lebanon, NH, USA) [27], which incorporated the national model.

### 2.3. Model Validation

Model validity was assessed using the following three methods: First, the model fit to the reference data was evaluated for each reference datum by sex and age in the national model. The mean absolute percentage error (MAPE) [20] was calculated for the daily salt intake of the fixed hypothetical population in the salt intake sub-model, population and deaths in the population sub-model, PwH in the hypertension sub-model, and treatment cost in the treatment cost sub-model by sex and age group. Second, the model parameters were compared between men and women, and across age groups, to confirm the absence of significant differences. Third, simulated results by sex and age group were compared between the national and prefectural models to assess the degree of similarity in their behaviors.

### 2.4. Sensitivity Analysis

A sensitivity analysis was conducted using Monte Carlo multivariate sensitivity testing [22]. A sample size of 200 simulations was generated; parameter variations at 0th, 5th, 25th, 50th, 75th, 95th, and 100th percentile bounds were calculated. A change between 80% and 120% of the parameters in the salt intake sub-model was applied to the daily salt intake, PwH, and treatment cost across the two parameter sets. The first set consisted of four parameters: initial low-salt foods, initial low-salt people, rate of change in salt content, and rate of change in salt intake. The second set consisted of six parameters: high-salt food level, low-salt food level, and the four parameters of the first set. In addition, a change from 1.4 to 1.8 in the multiplier of 1.6 for deaths among PwH by sex and age group in the hypertension sub-model was applied to PwH and treatment cost.

### 2.5. Scenarios

Using national and subnational models, simulations were conducted for the period 2012 to 2040 as the base run. In addition, three hypothetical scenarios were designed to simulate the effects of multiplying the change rates of food and people on daily salt intake, the number of PwH, and outpatient treatment costs for hypertension. A multiplier of three was chosen to indicate extreme changes.

- Scenario 1: The change rate of the foods multiplied by three.
- Scenario 2: The change rate of the people multiplied by three.
- Scenario 3: The change rates of both foods and people multiplied by three.

## 3. Results

### 3.1. Model Validation

The simulated results showed satisfactory agreement with the reference data. The model parameters were not substantially different between men and women or across age groups. The simulated results by sex and age group were similar across the national and prefectural models.

The MAPE was 1.0% for the daily salt intake of the fixed hypothetical population and less than 10% for population, deaths, and treatment costs by sex and age group (Table 1). The MAPE for PwH was approximately 20% for men and women in their 40s, and approximately 15% for men and women in their 60s.

### 3.2. Sensitivity Analysis

**Figure 3:**
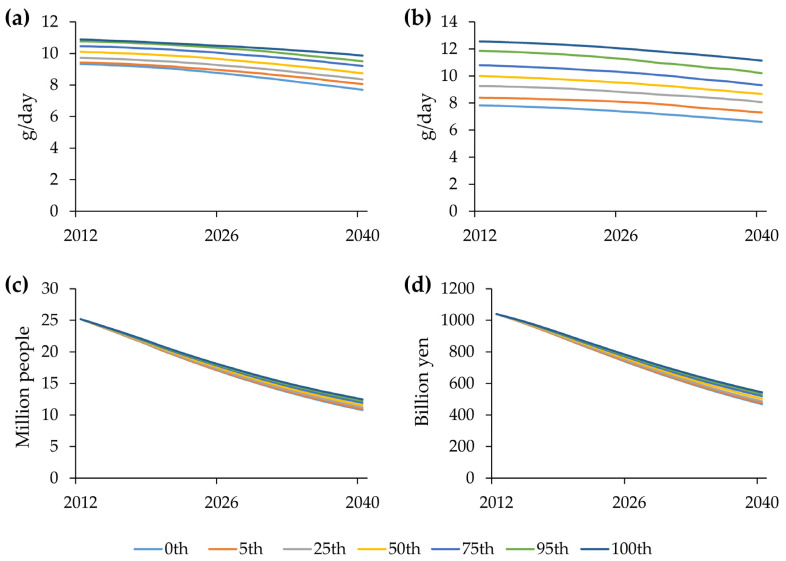
Simulated changes of 0th, 5th, 25th, 50th, 75th, 95th, and 100th percentile bounds in Monte Carlo multivariate sensitivity analyses based on a sample of 200 simulations with parameter values varied between 80% and 120% in the salt intake sub-model. Results are shown for daily salt intake (**a**) using the initial low-salt foods, the initial low salt people, the change rate of salt content, and the change rate of salt intake (the first set of parameters), and for daily salt intake (**b**), the number of PwH (**c**), and treatment cost (**d**) using the high-salt food level, the low-salt food level, the initial low-salt foods, the initial low salt people, the change rate of salt content, and the change rate of salt intake (the second set of parameters). PwH, people with hypertension.

The range between the 0th and 100th percentile bounds was wider under multivariate changes in the second set of parameters for daily salt intake (Figure 3b) than under multivariate changes in the first set of parameters (Figure 3a). Under multivariate changes in the second set of parameters, the simulated results for the 0% and 100% bounds of PwH and treatment cost in 2040 were 93.1% and 107.7% for PwH (Figure 3c) and 93.0% and 107.8% for treatment cost (Figure 3d), respectively, relative to the base run. In addition, multivariate changes in the multiplier of 1.6 for deaths among PwH by sex and age group yielded simulated results of 98.2% and 101.7% for PwH and 97.7% and 102.3% for treatment cost at the 0% and 100% bounds in 2040, respectively, compared with the base run.

### 3.3. Simulated Results of the National Model as Base Run

**Figure 4:**
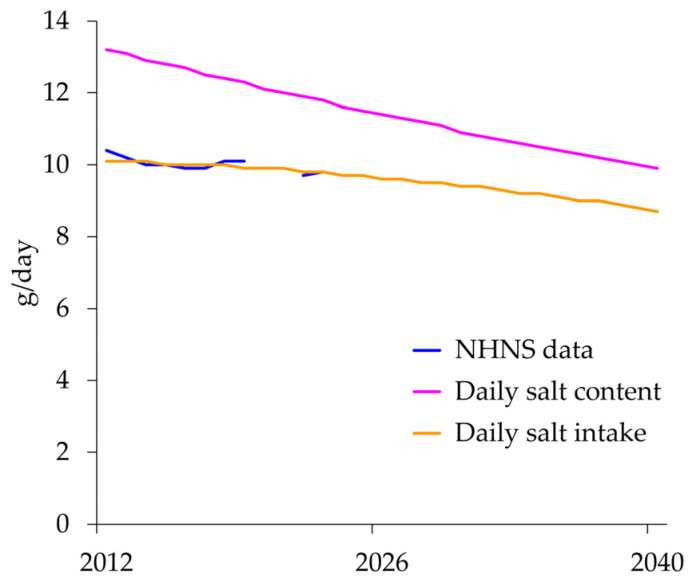
Daily salt intake in the NHNS (2012–2019, 2022, and 2023) and simulated changes in daily salt content and daily salt intake in the salt intake sub-model from 2012 to 2040 in the base run. NHNS, National Health and Nutrition Survey.

In the salt intake sub-model, the initial proportion of people with low salt intake and the rate of change were determined by optimization to be 48.7% and 0.0786 per year, respectively. As a result of a decrease in the number of high-salt foods and people with high salt intake, the daily salt content of the foods would decrease from 13.2 g/day in 2012 to 9.9 g/day in 2040 (Figure 4), and daily salt intake would decrease from 10.1 g/day in 2012 to 8.7 g/day in 2040 (Table 2).

**Figure 5:**
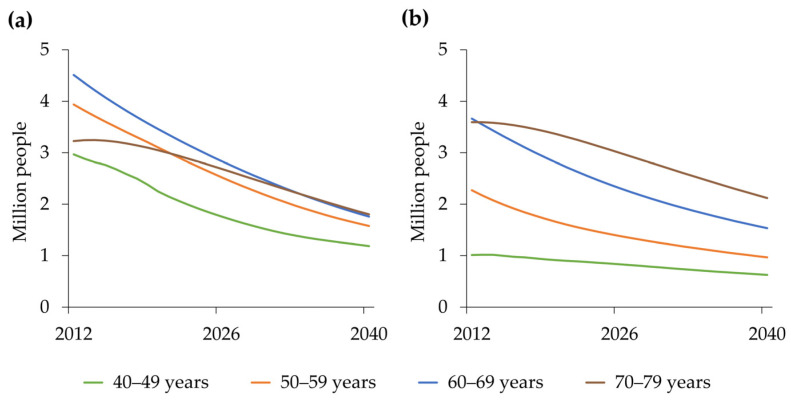
Simulated changes in the number of PwH by age group from 2012 to 2040 in the base run for men (**a**) and women (**b**). PwH, people with hypertension.

In the population sub-model, both men and women declined, and the decline was steeper for women. In the hypertension and treatment cost sub-models, both men and women declined, and the decline was steeper for men. In the hypertension and treatment cost sub-models, both men and women declined, and the decline was steeper for men. Simulated changes in PwH in men in their 40s and 50s showed a steeper decline from 2012 to 2040 than that in women (Figure 5).

### 3.4. Simulated Results of the National Model by Scenario

In the salt intake sub-model, scenario 1 had a larger impact on high-salt foods than scenario 2. Scenario 2 had a greater impact on individuals with high salt intake and daily salt intake than scenario 1, and scenario 3 had the greatest impact on these variables (Table 3).

In the hypertension and treatment cost sub-models, scenario 2 had a larger impact on PwH and treatment costs than scenario 1, whereas scenario 3 had the largest impact on these variables in both men and women. The differences relative to the base run were greater among women than among men, with respect to both PwH and treatment costs.

### 3.5. Simulated Results of Subnational Models

The daily salt intake in 2012 ranged from 8.3 g/day in Okinawa to 11.7 g/day in Iwate, and these values decreased to 6.9 g/day and 10.3 g/day in the base run, respectively (Table 4). A more than 10-fold difference was observed between Tottori and Tokyo in terms of population, PwH, and treatment costs. The prefecture models were simulated for each scenario, yielding the corresponding results.

## 4. Discussion

To our knowledge, this is the first study to use a national- and prefectural-level system dynamics model to assess the impact of reduced salt intake on medical expenditures for hypertension in Japan. Based on the national model, prefectural models were developed using each prefecture’s population sub-model, and the numbers of PwH and outpatient treatment costs for hypertension were simulated for the base run and the three scenarios by prefecture. The methodology for developing subnational models based on the national model used in this study may be applied to other public health interventions for chronic diseases.

Because the Japanese population shares a common food environment, with 70% of its dietary salt from seasonings [2], the daily salt intake was calculated for a fixed population of 100,000 people, and the proportion was applied to national and prefectural populations. In 2012, only 10% of 1000 foods were arbitrarily classified as low-salt. This means that the weighted mean of 90% of the foods weighing 14 g (high-salt foods) and 10% of the foods weighing 6 g (low-salt foods) was 13.2 g in 2012. The discrepancy in the daily salt intake of 10.4 g in 2012 [21] could be explained by the abundant availability of seasonings in supermarkets, convenience stores, and restaurants. As shown in the simulated changes in daily salt intake using reference data from the NHNS (Figure 4), the daily salt content closely matched the reference data, with an MAPE of 1.0%. In addition, the daily salt content of the foods decreased by 25% from 13.2 g/day in 2012 to 9.9 g/day in 2040, whereas the daily salt intake decreased by 14% from 10.1 g/day in 2012 to 8.7 g/day in 2040. Thus, the findings indicate that the daily salt content should be lowered more intensively than the daily salt intake. Sensitivity analyses showed that when the high-salt food level and the low-salt food level, which directly changed daily salt intake, were added to the first set of parameters, the bounds between 0th and 100th percentiles of simulated changes became wider for daily salt intake, but the bounds were within reasonable ranges of the simulated results in the base run in 2040: 14.6% (93.1–107.7%) for PwH and 14.8% (93.0–107.8%) for treatment cost. In addition, the sensitivity analyses showed that the initial low-salt foods (10% of the total foods) in the first set of parameters did not affect PwH and treatment cost materially.

In the base run from 2012 to 2040, PwH and outpatient treatment costs for hypertension were found to decrease by approximately 50% owing to reductions in daily salt intake and population size. In the age range of 40–79 years, the population distribution across age groups was similar between sexes: 25.9%, 23.3%, 28.4%, and 22.4% for men and 27.9%, 24.4%, 28.2%, and 19.5% for women in their 40s, 50s, 60s, and 70s, respectively. However, the largest population (peak) was observed later in men than in women in most age groups: 2016, 2020, 2012, and 2024 in men and 2015, 2012, 2012, and 2019 in women in their 40s, 50s, 60s, and 70s, respectively. Thus, the difference in the population between 2012 and 2040 is larger in women than in men.

In this study, hypertension was defined as systolic blood pressure ≥ 140 mmHg and/or diastolic blood pressure ≥ 90 mmHg, regardless of antihypertensive medication use, rather than as PwH controlled with antihypertensive medication. This is because the number of patients taking antihypertensive medication is determined by a combination of factors, including the prevalence of high blood pressure, prescribing by medical doctors, and the affordability of medication. This complicates the model; therefore, we obtained outpatient treatment costs for hypertension per person by calibrating medical expenditures by sex and age group. We acknowledge that this remains a challenge for future model improvement. Another reason was that the focus of this study was to examine the impact of salt reduction as a lifestyle modification on medical expenditure for hypertension rather than promoting antihypertensive medication use without modifying lifestyle. Based on this definition, the inflow for the development of hypertension in the hypertension sub-model was calculated by multiplying the proportion of people with high salt intake. When the parameters of this inflow were compared by sex and age group, the parameters for men were consistently lower than those for women, particularly for men in their 60s, who were negative (−0.0035 for men and 0.0104 for women in their 60s). This sex difference seemed to have contributed to the differences in the number of PwH and treatment costs between men and women in each scenario. We acknowledge that this framework is based on correlation rather than causation [20]. However, to simplify the model, we relied on the well-established relationship between high salt intake and hypertension development [9]. This modeling approach has potential policy implications for population-level salt reduction, as it illustrates that decreasing the proportion of individuals with high salt intake may reduce the number of PwH. This remains a crucial challenge for the development of future models.

In addition, 1.6 was used as a multiplier for the outflows of deaths from the respective stocks, and this value fell within the range of relative risks for blood pressure categories [25]. When this value was changed from 1.4 to 1.8 for each sex and age group, the calibrated inflow parameters did not change substantially. We also performed sensitivity analysis for simulated changes by varying the multiplier of 1.6 between 1.4 and 1.8. The simulated results for 0% and 100% bounds of PwH and treatment cost remained within a narrow range of the simulated results in the base run in 2040: 3.5% (98.2–101.7%) for PwH and 4.6% (97.7–102.3%) for treatment cost. The MAPE for PwH was approximately 20% for men and women in their 40s, and approximately 15% for men and women in their 60s. This could be attributed to the strong assumption that prevalence data from 2023 apply to 2040, especially for men and women in their 40s. The effects of such a strong assumption would be rather small, as the outpatient treatment costs for hypertension per person were smaller in younger age groups: 12,344.4, 21,758.6, 44,145.7, and 65,286.6 for men; and 17,267.6, 28,016.3, 47,716.0, and 70,117.7 for women in their 40s, 50s, 60s, and 70s, respectively.

This study used the change rates of food and population to examine the impact of salt reduction on medical expenditure for hypertension but did not specify measures for each change [18]. Our previous study examined the health and economic effects of salt-reduction interventions to prevent noncommunicable diseases in Japan and found that food-product reformulation had a greater impact than adopting a low-salt diet. The current study employed a simplified model to focus on hypertension and various scenarios; however, the importance of reformulating food products should also be emphasized to accelerate food changes.

This study has several limitations. First, hypertension is a risk factor for chronic diseases, such as cardiovascular and chronic kidney diseases [9], and the treatment of these diseases greatly contributes to medical expenditure in Japan; this study exclusively focused on hypertension. A comprehensive model of public health interventions for chronic diseases, such as PRISM in the United States [15,16], should be developed by expanding the current model. Second, although deaths due to hypertension were incorporated into the hypertension sub-model, changes in deaths according to scenarios were not fed back into the population sub-model. As mortality (per 100,000) from hypertensive diseases (9.8) only accounted for 0.7% of all-cause mortality (1334.5) in 2024 [24], the effects of this omission seem minor in the current model. However, hypertension is a risk factor for chronic diseases. This issue should be resolved when the current model is expanded to include cardiovascular and chronic kidney diseases. Third, the same parameters were used across all sex and age groups for the rate-of-change in the salt intake sub-model and for the hypertension sub-model’s ratio of PwH in each prefecture to that in Japan. This might have led to a lack of detailed simulations; however, it avoided providing extreme results based on rather small samples by sex and age group in the NHNS. Fourth, we applied identical structural parameters, such as change rates in the salt intake sub-model and mortality multipliers in the hypertension sub-model. This approach did not account for differences among prefectures in dietary culture, demographic trends, or healthcare access. A more flexible modeling approach would improve the applicability of subnational models.

## 5. Conclusions

In conclusion, the national and prefectural system dynamics models indicate that reducing salt intake could reduce PwH and treatment costs by 2040. These simulation models could help national and local government policymakers promote salt reduction to reduce medical expenditures in Japan.

## Figures and Tables

**Table 1:** MAPE (%) for the parameters in the population sub-model, the hypertension sub-model and the treatment cost sub-model by sex and age group.

	Men (Age, Years)				Women (Age, Years)			
	40–49	50–59	60–69	70–79	40–49	50–59	60–69	70–79
Population sub-model								
Population	2.0	1.3	2.8	2.6	3.9	1.4	2.7	2.5
Deaths	7.7	4.0	3.3	2.5	2.8	5.5	2.9	2.4
Hypertension sub-model								
PwH	23.9	7.0	13.0	8.4	19.2	8.9	15.7	5.7
Treatment cost sub-model								
Treatment cost	6.7	4.5	3.0	2.8	2.6	2.2	4.5	2.5

MAPE, mean absolute percentage error; PwH, people with hypertension.

**Table 2:** Simulated results of the national model as base run.

	2012	2026	2040	Difference from 2012 to 2040 (%)
Salt intake sub-model				
Number of high-salt foods	900	676	483	−46.3
Number of People with high salt intake	51,313	45,415	34,322	−33.1
Daily salt intake, g/day	10.1	9.6	8.7	−13.9
Population sub-model				
Number of men, million	31.6	30.8	27.7	−12.3
Number of women, million	33.2	30.8	26.5	−20.2
Hypertension sub-model				
Number of PwH men, million	14.6	9.8	6.3	−56.8
Number of PwH women, million	10.5	7.5	5.2	−50.5
Treatment cost sub-model				
Treatment cost for men, billion yen	531.9	377.5	244.3	−54.1
Treatment cost for women, billion yen	507.8	373.8	259.8	−48.8

PwH, people with hypertension.

**Table 3:** Simulated results of the national model by scenario.

	Simulated Results in 2040				Difference from Base Run in 2040, %		
	Base Run	Scenario 1	Scenario 2	Scenario 3	Scenario 1	Scenario 2	Scenario 3
Salt intake sub-model							
Number of high-salt foods	483	242	459	201	−50.0	−5.1	−58.5
Number of people with high salt intake	34,322	30,793	16,652	10,639	−10.3	−51.5	−69.0
Daily salt intake, g/day	8.7	8.5	7.3	6.9	−3.2	−16.2	−21.7
Hypertension sub-model							
Number of PwH men, million	6.3	6.3	6.2	6.2	−0.3	−1.7	−2.3
Number of PwH women, million	5.2	5.2	4.9	4.8	−1.0	−6.6	−8.8
Treatment cost sub-model							
Treatment cost for men, billion yen	244.3	243.7	240.6	239.3	−0.2	−1.5	−2.0
Treatment cost for women, billion yen	259.8	257.4	243.7	238.3	−0.9	−6.2	−8.3

PwH, people with hypertension.

**Table 4:** Minimum and maximum simulated results from subnational models in the base run.

	2012	2026	2040	Prefecture
Minimum (2012)				
Daily salt intake, g/day	8.3	7.8	6.9	Okinawa
Population, million	0.30	0.28	0.23	Tottori
Number of PwH, million	0.14	0.09	0.06	Tottori
Treatment cost, billion yen	5.8	4.0	2.5	Tottori
Maximum (2012)				
Daily salt intake, g/day	11.7	11.2	10.3	Iwate
Population, million	6.58	6.47	5.98	Tokyo
Number of PwH, million	1.82	1.41	1.06	Tokyo
Treatment cost, billion yen	72.3	58.4	44.3	Tokyo

PwH, people with hypertension.

## Data Availability

Restrictions apply to the availability of these data. Data were obtained from MHLW and are available with the permission of MHLW under the Statistics Act.

## Abbreviations

The following abbreviations are used in this manuscript:

MAPE	mean absolute percentage error
MHLW	Ministry of Health, Labor and Welfare
NHNS	National Health and Nutrition Survey
PwH	people with hypertension
